# Development of a predictive nomogram based on preoperative inflammation-nutrition-related markers for prognosis in locally advanced lip squamous cell carcinoma after surgical treatment

**DOI:** 10.1186/s12903-025-05663-6

**Published:** 2025-02-20

**Authors:** Hao Cheng, Xue-Lian Xu, Zheng Zhang, Jin-Hong Xu, Zhuo-Ran Li, Ya-Nan Wang, Bo-Dong Zhang, Ke Chen, Shou-Yu Wang

**Affiliations:** 1 Department of Radiotherapy Oncology, The First Affiliated Hospital of Xinxiang Medical University, Xinxiangaq, 453100 Henan China; 2 Department of Radiotherapy Oncology, Affiliated Cancer Hospital of Zhengzhou University, Zhengzhouaq, 450000 Henan China; 3 Department of Otolaryngology, AnYang District Hospital, Anyang, 455000 Henan China; 4 Department of Medical Oncology, The First Affiliated Hospital of Xinxiang Medical University, Xinxiangaq, 453100 Henan China; 5 Department of Student Affairs, The Second Affiliated Hospital of Qiqihar Medical University, Qiqihar, 161000 Heilongjiang China

**Keywords:** Lip squamous cell carcinoma, Inflammation-nutrition-related markers, Geriatric nutritional risk index, Controlling nutrition score, Prognosis

## Abstract

**Background:**

The prognostic role of preoperative inflammation-nutrition-related markers in locally advanced lip squamous cell carcinoma (LSCC) remains underexplored. This study aimed to assess the impact of various preoperative inflammation-nutrition-related markers on the prognosis of patients with locally advanced LSCC undergoing surgical treatment and to establish a corresponding predictive model.

**Methods:**

A retrospective analysis was performed on the clinical data of 169 patients with locally advanced LSCC who underwent surgical treatment. A total of 27 clinicopathological variables, including inflammation-nutrition-related markers, were collected. Univariate and multivariate Cox regression analyses were used to identify independent prognostic factors for disease-free survival (DFS) and overall survival (OS). The nomogram models were validated using receiver operating characteristic (ROC) curve analysis, calibration plots, and decision curve analysis (DCA). Risk stratification was performed based on the nomogram scores, and differences between risk subgroups were explored.

**Results:**

The extranodal extension (ENE), surgical safety margin, Glasgow prognostic score (GPS), Geriatric Nutritional Risk Index (GNRI), Controlling Nutrition score (CONUT), American Joint Committee on Cancer (AJCC) stage, and adjuvant radiotherapy were independent prognostic factors for DFS. In contrast, ENE, surgical safety margin, GNRI, CONUT, AJCC stage, and adjuvant radiotherapy were also independent prognostic factors for OS. The nomograms demonstrated better predictive performance than the AJCC staging system. Based on the nomogram model, patients were stratified into low-, medium-, and high-risk subgroups, which exhibited significant differences in survival outcomes.

**Conclusion:**

GPS, GNRI, and CONUT are independent factors affecting the prognosis of patients with locally advanced LSCC undergoing radical surgery. By combining GPS, GNRI, and COUNT with other independent clinicopathological prognostic factors, a reliable nomogram model can be established to accurately predict patients' DFS and OS. This provides a powerful tool for individualized prognostic assessment, optimized risk stratification, and treatment decision-making.

**Supplementary Information:**

The online version contains supplementary material available at 10.1186/s12903-025-05663-6.

## Introduction

The incidence of oral cancer is projected to constitute approximately 2% of all cancer cases in 2022, while the mortality rate is expected to account for about 1.9% of all cancers [1]. The predominant histological subtype of lip carcinoma is squamous cell carcinoma, accounting for more than 25% of all cases of oral cancer [2]. The results of previous retrospective analyses have consistently demonstrated that patients diagnosed with locally advanced lip squamous cell carcinoma (LSCC) or those presenting with distant metastasis exhibit a significantly poorer prognosis compared to individuals in the early stages cases [3–5]. The exposure factors that increase the risk of LSCC include smoking, alcohol consumption, genetic predisposition, sun exposure, and immunocompromised status [6]. Surgical intervention is regarded as the primary therapeutic approach for managing LSCC [2, 7].

Currently, the AJCC staging system remains the primary tool for predicting the prognosis of LSCC, with its latest version being the 8th edition [8]. However, this system fails to encompass numerous critical prognostic factors. The objective of this study is to explore additional potential prognostic variables that may provide more comprehensive information. Among the many factors affecting tumor prognosis, inflammation-nutrition-related markers have recently gained attention. These markers encompass various indices [9–15], such as the neutrophil-to-lymphocyte ratio (NLR), lymphocyte-to-monocyte ratio (LMR), platelet-to-albumin ratio (PAR), platelet-to-lymphocyte ratio (PLR), prognostic nutrition index (PNI), systemic inflammation score (SIS), Glasgow prognostic score (GPS), Geriatric Nutritional Risk Index (GNRI), and Controlling Nutritional status (CONUT) among others. A body of research has consistently demonstrated the prognostic significance of inflammation and nutrition-related biomarkers in oral carcinoma [16–18]. However, the impact of these inflammation-nutrition-related markers on the prognosis of patients with locally advanced LSCC remains uncertain. Moreover, despite previous studies developing survival prediction models for lip cancer [5, 19], there is currently no prognostic model available for locally advanced LSCC that incorporates a comprehensive range of inflammation-nutrition-related markers. Therefore, this study serves as a valuable addition to existing research.

Building on this, our study aims to investigate the correlation between various inflammation-nutrition-related markers and the prognosis of locally advanced LSCC. Additionally, we plan to develop a nomogram model and assess its superiority over the AJCC staging system in prognostic prediction.

## Method

### Cases selection

**Fig. 1:**
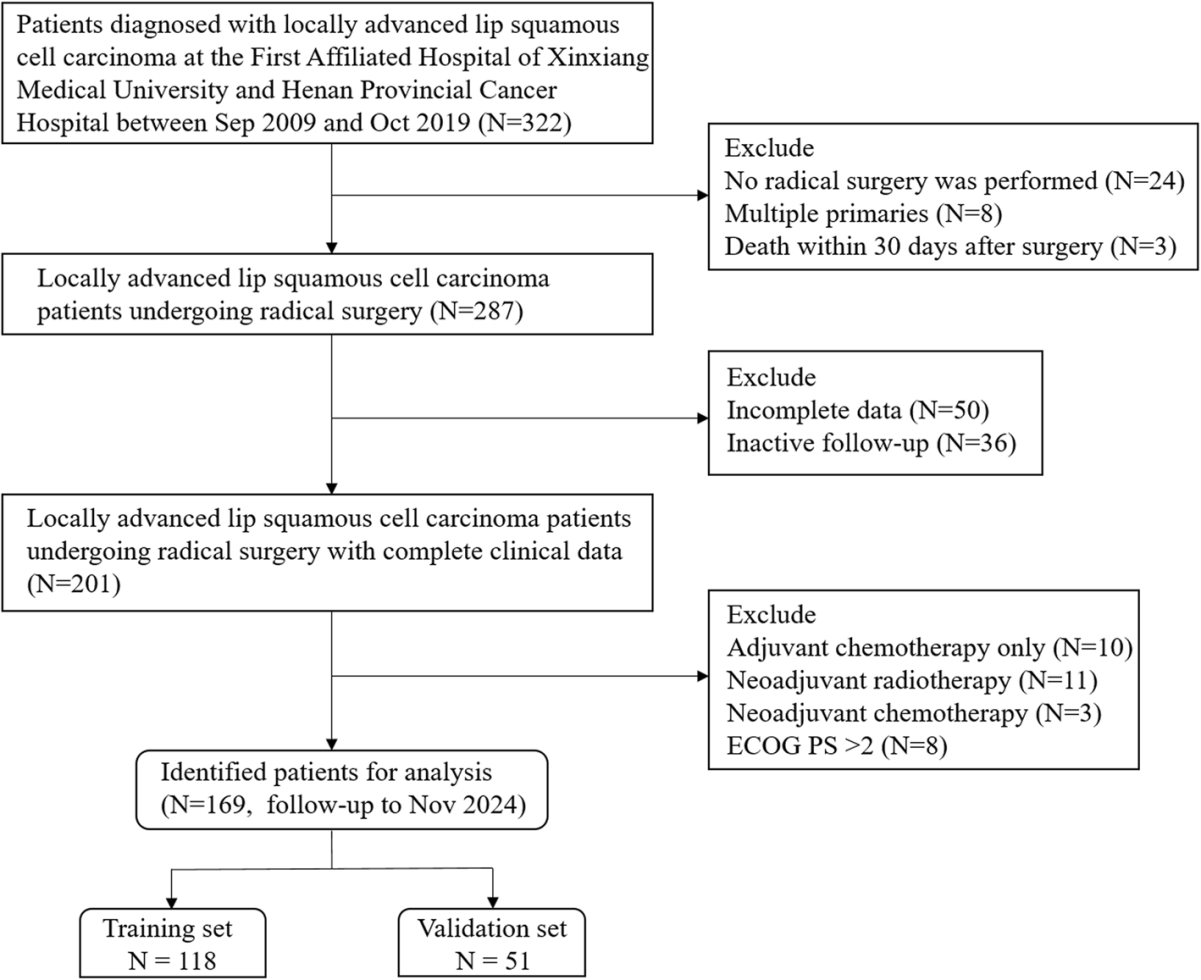
The flow chart for the study. ECOG, eastern cooperative oncology group; PS, performance status

A total of 169 patients diagnosed with locally advanced LSCC were enrolled in this study. The inclusion criteria were as follows: (1) histopathologically confirmed LSCC; (2) clinical stage III, and IVa—IVb according to the AJCC 8th edition; (3) age ≥ 18. Exclusion criteria included: (1) patients who did not undergo radical surgery; (2) presence of distant metastasis at initial diagnosis; (3) death within 30 days after surgery; (4) missing or incomplete follow-up data; (5) presence of multiple primary tumors; (6) incomplete clinical baseline data; (7) patients who received postoperative adjuvant chemotherapy only; (8) receiving neoadjuvant chemotherapy or neoadjuvant radiotherapy; (9) receiving immunotherapy, and (10) eastern cooperative oncology group (ECOG) performance status (PS) score > 2. The study protocol underwent a thorough review and received approval from the institutional ethics committee. Prior to their participation in this study, all enrolled patients provided explicit and informed consent. The employed radiotherapy techniques in this research included conformal radiotherapy (CRT), intensity-modulated radiotherapy (IMRT), and volumetric modulated arc therapy (VMAT). A total dose of 60.0–70.0 Gy was administered, fractionated with a dose of 1.96–2.18 Gy per fraction, once daily for five days each week. The detailed flow chart is illustrated in Fig. 1. In this study, adjuvant chemotherapy as a variable refers to chemotherapy administered concurrently with adjuvant radiotherapy. The chemotherapy drugs include platinum-based agents and taxanes.

### Clinicopathological factors

A total of 30 clinicopathological variables were included in this study, including gender, age at diagnosis, tumor location, differentiation, depth of invasion (DOI), smoking, extranodal extension (ENE), surgical safety margin, perineural invasion, vascular invasion (VI), ECOG PS, total cholesterol (TC), hemoglobin, albumin, lymphocyte count, NLR, LMR, PAR, PLR, PNI, body mass index (BMI), SIS, GPS, GNRI, CONUT, American Joint Committee on Cancer (AJCC) Stage, adjuvant radiotherapy, chemotherapy, disease-free survival (DFS), and overall survival (OS). The assessments for TC, hemoglobin, albumin, lymphocyte count, NLR, LMR, PAR, PLR, PNI, BMI, SIS, GPS, GNRI, and CONUT were all conducted within a week prior to the surgical procedure. The study endpoints included DFS and OS. DFS was defined as the duration from completion of treatment to the occurrence of disease recurrence or progression, while OS referred to the period from initiation of treatment to death resulting from any cause. In order to facilitate interpretation and clinical application, DOI and surgical safety margin were transformed into binary variables by X-tile software, and the cut-off values were 10 mm and 5 mm, respectively.

### Calculations

The calculation formula for CONUT is presented in Table S1. The calculation formulas for NLR, LMR, PAR, PLR, PNI, BMI, SIS, GPS, and GNRI are detailed in Table S2. The optimal cut-off point of GNRI was determined to be 94.2 using X-tile software.

### Analysis

SPSS 20.0 and R 4.22 software were utilized for data analysis. Patients were randomly assigned to either the training set or the validation set in a 7:3 ratio. To evaluate differences in all variables between these two sets, we used an independent sample t-test for continuous variables and the Chi-square test for categorical variables. The training set was employed for both univariate and multivariate Cox regression analyses, as well as for constructing nomograms. Conversely, the validation set was used for a series of checks.

To assess multicollinearity, we calculated the variance inflation factor (VIF) for all included variables. The VIF is calculated in SPSS software using linear regression analysis under the Collinearity Diagnostics option. The results indicated that the VIF values for BMI, GNRI, albumin, lymphocyte count, GPS, and PNI were 11.221, 10.645, 10.147, 9.660, 7.100, and 6.578, respectively. To address this issue, we excluded BMI, lymphocyte count, and albumin from the Cox regression model. After recalculating the VIF for the remaining variables, all values were below 5 (Table S3), suggesting that multicollinearity was no longer a concern. This approach was intended to mitigate any multicollinearity issues.

Subsequently, we included the remaining variables in the univariable Cox regression analysis to identify potential factors influencing DFS and OS. Potential prognostic factors were then included in a multivariable Cox regression analysis to determine their independence as prognostic indicators. To further reduce potential confounding effects, we also incorporated some clinically significant variables into the multivariable Cox regression, This approach was intended to mitigate any multicollinearity issues. The backward stepwise selection method was applied in the multivariable Cox regression analysis to refine the final models. Ultimately, these independent prognostic factors were integrated into two separate nomograms designed to predict DFS and OS, respectively.

### Validation

The validation process was conducted using R software, version 4.2.2. To comprehensively assess the predictive capability of the nomogram, we employed multiple methodologies including receiver operating characteristic (ROC) curves, calibration plots, and decision curve analysis (DCA). The area under the ROC curve (AUC) was utilized to quantify the model's accuracy in predicting DFS and OS. A higher AUC indicates a stronger predictive power of the model. Calibration plots were employed to compare predicted outcomes with actual results, thereby confirming the model's reliability. DCA evaluated the clinical utility of the model at different threshold probabilities and guided physicians in treatment decision-making processes. Additionally, we calculated the Integrated Discrimination Improvement Index (IDI) and Net Reclassification Improvement Index (NRI) to compare performance between our nomogram and conventional AJCC staging system. These validation processes help elucidate improvements in classification and prediction accuracy offered by our nomogram, providing insights into its potential advantages.

### Establishment of risk stratification system

Each independent prognostic variable is assigned a corresponding score in the nomogram, and these points are then summed to calculate the total points. The X-tile software was utilized to determine the optimal cut-off value for the total points. Based on this cut-off value, patients were categorized into low, medium, and high-risk groups according to their respective total points. The survival rates of patients in different risk subgroups were analyzed using the Kaplan–Meier method, and corresponding Kaplan–Meier curves were generated.

## Results

### Baseline characteristics

After applying the inclusion and exclusion criteria, a total of 169 patients were selected for the final analysis. The detailed documentation of all patient baseline characteristics can be found in Table 1.

**Table 1:** The baseline characteristics of postoperative locally advanced LSCC patients and the difference between the two cohorts

Characteristics	All Patients (*n* = 169) *n* (%)	Training cohort (*n* = 118) *n* (%)	Validation cohort (*n* = 51) *n* (%)	*P*
**Gender**				0.836
Male	61 (36.1%)	42 (35.6%)	19 (37.3%)	
Female	108 (63.9%)	76 (64.4%)	32 (62.7%)	
**Age at diagnosis (years)**				0.561
Median (IQR)	51 (38–69)	52 (39–69)	49 (35–67)	
**Tumor location**				0.513
Lower	141 (83.4%)	97 (82.2%)	44 (86.3%)	
Upper	28 (16.6%)	21 (17.8%)	7 (13.7%)	
**Differentiation**				0.538
Well	57 (33.7%)	39 (33.1%)	18 (35.3%)	
Moderate	73 (43.2%)	49 (41.5%)	24 (47.1%)	
Poor	39 (23.1%)	30 (25.4%)	9 (17.6%)	
**DOI**				0.706
< 10 mm	122 (72.2%)	84 (71.2%)	38 (74.5%)	
≥ 10 mm	47 (27.8%)	34 (28.8%)	13 (25.5%)	
**Smoking**				0.628
No	152 (76.3%)	107 (90.7%)	45 (88.3%)	
Yes	17 (23.7%)	11 (9.3%)	6 (11.7%)	
**ENE**				0.590
Negative	117 (69.2%)	82 (69.5%)	35 (68.6%)	
Positive	52 (30.8%)	36 (30.5%)	16 (31.4%)	
**Surgical safety margin**				0.714
≥ 5 mm	129 (76.3%)	91 (77.1%)	38 (74.5%)	
< 5 mm or positive	40 (23.7%)	27 (22.9%)	13 (25.5%)	
**Perineural invasion**				0.736
No	139 (82.2%)	96 (81.4%)	43 (84.3%)	
Yes	30 (17.8%)	22 (18.6%)	8 (15.7%)	
**VI**				0.779
No	137 (81.1%)	95 (80.5%)	42 (68.6%)	
Yes	32 (18.9%)	23 (19.5%)	9 (31.4%)	
**ECOG PS score**				0.135
0–1	131 (77.5%)	90 (76.3%)	41 (80.4%)	
2	38 (22.5%)	28 (23.7%)	10 (19.6%)	
**TC**				0.931
Median (IQR)	193.9 (134.5–260.2)	193.0 (134.8–258.5)	197.3 (130.3–263.0)	
**Hemoglobin (g/L)**				0.566
Median (IQR)	100.5 (92.2–121.1)	100.0 (92.2–121.1)	103.0 (91.1–122.2)	
**Albumin (g/L)**				0.742
Median (IQR)	41.0 (34.0–48.3)	41.5 (34.0–48.3)	39.5 (36.6–48.9)	
**Lymphocyte Count** (10^9^/L)				0.237
Median (IQR)	1.97 (1.09–2.95)	1.98 (0.93–2.95)	1.88 (1.26–3.03)	
**NLR**				0.562
Median (IQR)	2.25 (1.45–3.19)	2.48 (1.60–2.28)	1.77 (1.09–2.83)	
**LMR**				0.906
Median (IQR)	4.99 (2.42–7.43)	5.12 (2.47–7.41)	4.71 (2.08–7.96)	
**PAR**				0.800
Median (IQR)	7.59 (4.13–10.06)	8.12 (4.54–10.46)	6.94 (3.86–9.17)	
**PLR**				0.502
Median (IQR)	148.5 (88.1–203.5)	154.0 (93.8–208.5)	124.5 (82.0–190.5)	
**PNI**				0.497
Median (IQR)	73.0 (54.4–93.2)	70.5 (53.2–91.3)	80.4 (55.4–99.3)	
**BMI**				0.484
Median (IQR)	21.3 (19.5–25.0)	21.0 (19.6–24.9)	23.5 (19.6–26.1)	
**SIS**				0.533
0	121 (71.6%)	82 (69.5%)	39 (76.5%)	
1	28 (16.6%)	20 (16.9%)	8 (15.7%)	
2	20 (11.8%)	16 (13.6%)	4 (7.8%)	
**GPS**				0.499
0	113 (66.9%)	77 (65.3%)	36 (70.6%)	
1–2	56 (33.1%)	41 (34.7%)	15 (29.7%)	
**GNRI**				0.431
≥ 94.2	95 (56.2%)	64 (54.2%)	31 (60.8%)	
< 94.2	74 (43.8%)	54 (45.8%)	20 (39.2%)	
**CONUT**				0.845
< 2	98 (58.0%)	69 (58.5%)	29 (56.9%)	
≥ 2	71 (42.0%)	49 (41.5%)	22 (43.1%)	
**AJCC Stage**				0.279
III	100 (59.2%)	73 (61.9%)	27 (52.9%)	
IVa / IVb	69 (40.8%)	45 (38.1%)	24 (47.1%)	
**Adjuvant radiotherapy**				0.479
No	46 (27.2%)	34 (28.8%)	12 (23.5%)	
Yes	123 (72.8%)	84 (71.2%)	39 (76.5%)	
Adjuvant chemotherapy*				0.230
No	117 (69.2%)	85 (72.0%)	32 (62.7%)	
Yes	52 (30.8%)	33 (28.0%)	19 (37.3%)	
**DFS (months)**				0.537
Median (range)	28 (3–129)	27 (3–125)	29 (7–129)	
**OS (months)**				0.863
Median (range)	46 (3–135)	46 (3–126)	47 (7–135)	

*Abbreviations: AJCC* American Joint Committee on Cancer, *BMI* body mass index, *CI* confidence interval, *CONUT* controlling nutrition score, *DOI* depth of invasion, *DFS* disease-free survival, *ECOG PS* eastern cooperative oncology group performance status, *ENE* extranodal extension, *GNRI* Geriatric Nutritional Risk Index, *GPS* Glasgow prognostic score, *HR* hazard ratio, *IQR* Interquartile Range, *LSCC* lip squamous cell carcinoma, *LMR* lymphocyte-to-monocyte ratio, *NLR* neutrophil-to-lymphocyte ratio, *PAR* platelet-to-albumin ratio, *PLR* platelet-to-lymphocyte ratio, *PNI* prognostic nutrition index, *SIS* systemic inflammation score, *TC* total cholesterol, *VI* vascular invasion

^*^Refers to chemotherapy administered concurrently with radiotherapy

The majority of patients were male (*N* = 108, 63.9%). The median age was 51 years (Interquartile Range (IQR) 38–69). The primary site of 83.4% of cases (*N* = 141) was the lower lip. The number of cases with a DOI ≥ 10 mm, ENE, perineural invasion, VI, and surgical safety margin < 5 mm or positive was 47, 52, 30, 32, and 40 respectively. 131 (77.5%) patients exhibited an ECOG PS score of 0 or 1. The median value of TC was 193.9 (mg/dl), with an IQR of 134.5–206.2 mg/dl. The median PNI was 73.0, with an IQR of 54.4–93.2. There were 121 cases with an SIS of 0, 28 cases with an SIS of 1, and 20 cases with an SIS of 2, accounting for 71.6%, 16.6% and 11.8% of the total cases, respectively. The GNRI ≥ 94.2 was observed in 95 patients, accounting for 56.2% of the total. The CONUT < 2 in 98 patients (59.2%). In terms of AJCC staging, 59.2% of the patients (*N* = 100) were stage III and 40.8% of the patients (*N* = 69) were stage IVa to IVb. A total of 123 patients (72.8%) underwent adjuvant radiotherapy, while only 52 patients (30.8%) received adjuvant chemotherapy.

The median duration of DFS and OS were 28 months and 46 months, respectively. Additionally, the differences in each variable between the training set and validation set are presented in Table 1. The results indicate that there were no statistically significant differences observed for any of the variables between the two sets (all* P* > 0.05).

### Identification of independent prognostic factors

The independent prognostic factors were identified through univariate and multivariate Cox regression analyses (Tables 2 and 3). The independent prognostic factors affecting DFS include ENE, surgical safety margin, GPS, GNRI, CONUT, AJCC staging system, and adjuvant radiotherapy. Similarly, the independent prognostic factors influencing OS are ENE, surgical safety margin, GNRI, CONUT, AJCC staging system, and adjuvant radiotherapy.

**Table 2:** Univariate and multivariate analyses of variables in postoperative locally advanced LSCC patients for DFS

Characteristics	Univariate analysis	*P*	Multivariate analysis	*P*
	HR (95% CI)		HR (95% CI)	
**Gender**				
Male	Reference			
Female	1.244 (0.696 – 2.223)	0.462		
**Age at diagnosis** (years)	1.018 (1.002 – 1.034)	**0.025**	1.010 (0.994 – 1.027)	0.219
**Tumor location**				
Lower	Reference			
Upper	1.547 (0.817 – 2.928)	0.180		
**Differentiation**				
Well	Reference		Reference	
Moderate	1.263 (0.681 – 2.343)	0.458	1.103 (0.538 – 2.261)	0.789
Poor	1.851 (0.951 – 3.605)	0.070	1.890 (0.886 – 4.031)	0.100
**DOI**				
< 10 mm	Reference		Reference	
≥ 10 mm	1.814 (1.021 – 3.223)	**0.042**	1.310 (0.886 – 2.409)	0.438
**Smoking**				
No	Reference		Reference	
Yes	1.736 (0.742 – 4.064)	0.203	1.073 (0.382 – 3.013)	0.893
**ECOG PS score**				
0–1	Reference		Reference	
2	1.966 (1.135 – 3.408)	**0.016**	1.296 (0.669 – 2.509)	0.443
**ENE**				
Negative	Reference		Reference	
Positive	3.297 (1.883 – 5.776)	**< 0.001**	2.461 (1.302 – 4.650)	**0.006**
**Surgical safety margin**				
≥ 5 mm	Reference		Reference	
< 5 mm or positive	2.639 (1.528 – 4.556)	**< 0.001**	2.225 (1.214 – 4.080)	**0.010**
**Perineural invasion**				
No	Reference		Reference	
Yes	1.947 (1.073 – 3.531)	**0.028**	1.623 (0.822 – 3.208)	0.163
**VI**				
No	Reference		Reference	
Yes	2.009 (1.088 – 3.712)	**0.026**	0.916 (0.405 – 2.072)	0.834
**TC**	0.998 (0.995 – 1.001)	0.249		
**Hemoglobin**	1.002 (0.992 – 1.013)	0.650		
**NLR**	0.962 (0.743 – 1.244)	0.767		
**LMR**	0.930 (0.852 – 1.016)	0.109		
**PAR**	0.980 (0.910 – 1.055)	0.591		
**PLR**	1.001 (0.998 – 1.003)	0.542		
**PNI**	0.990 (0.979 – 1.001)	0.077		
**SIS**				
0	Reference		Reference	
1	1.093 (0.567 – 2.107)	0.791	2.877 (1.428 – 5.799)	0.219
2	1.477 (1.035 – 4.307)	**0.040**	1.905 (0.851 – 4.267)	0.117
**GPS**				
0	Reference		Reference	
1–2	1.974 (1.143 – 3.407)	**0.015**	2.059 (1.128 – 3.748)	**0.019**
**GNRI**				
≥ 94.2	Reference		Reference	
< 94.2	2.412 (1.415 – 4.112)	**0.001**	3.091 (1.743 – 5.481)	**< 0.001**
**CONUT**				
< 2	Reference		Reference	
≥ 2	2.482 (1.443 – 4.268)	**0.001**	2.491 (1.356 – 4.575)	**0.003**
**AJCC stage**				
III	Reference		Reference	
IVa&b	3.652 (1.947 – 6.847)	**0.003**	2.465 (1.399 – 4.345)	**0.002**
**Adjuvant radiotherapy**				
No	Reference		Reference	
Yes	0.419 (0.236 – 0.745)	**0.003**	0.456 (0.239 – 0.872)	**0.018**
**Adjuvant chemotherapy**				
No	Reference			
Yes	0.819 (0.455 – 1.475)	0.507		

*Abbreviations: AJCC* American Joint Committee on Cancer, *CI* confidence interval, *CONUT* Controlling Nutrition scores, *DOI* depth of invasion, *DFS* disease-free survival, *ECOG PS* eastern cooperative oncology group performance status, *ENE* extranodal extension, *GNRI* Geriatric Nutritional Risk Index, *GPS* Glasgow prognostic score, *HR* hazard ratio, *LSCC* lip squamous cell carcinoma, *LMR* lymphocyte-to-monocyte ratio, *NLR* neutrophil-to-lymphocyte ratio, *PAR* platelet-to-albumin ratio, *PLR* platelet-to-lymphocyte ratio, *PNI* prognostic nutrition index, *SIS* systemic inflammation score, *TC* total cholesterol, *VI* vascular invasion

**Table 3:** Univariate and multivariate analyses of variables in postoperative LSCC patients for OS

Characteristics	Univariate analysis	*P*	Multivariate analysis	*P*
	HR (95% CI)		HR (95% CI)	
**Gender**				
Male	Reference			
Female	1.100 (0.600 – 2.017)	0.795		
**Age at diagnosis** (years)	1.020 (1.002 – 1.037)	**0.027**	1.011 (0.993 – 1.029)	0.241
**Tumor location**				
Lower	Reference			
Upper	1.935 (1.009 – 3.712)	0.047		
**Differentiation**				
Well	Reference		Reference	
Moderate	1.040 (0.534 – 2.026)	0.908	0.789 (0.374 – 1.663)	0.534
Poor	1.988 (0.986 – 4.007)	0.055	1.650 (0.761 – 3.578)	0.205
**DOI**				
< 10 mm	Reference			
≥ 10 mm	1.284 (0.689 – 2.393)	0.431		
**Smoking**				
No	Reference		Reference	
Yes	1.610 (0.637 – 4.072)	0.314	0.680 (0.201 – 2.302)	0.535
**ECOG PS score**				
0–1	Reference		Reference	
2	1.936 (1.066 – 3.516)	**0.030**	0.984 (0.437 – 2.215)	0.968
**ENE**				
Negative	Reference		Reference	
Positive	3.266 (1.766 – 6.038)	**< 0.001**	3.008 (1.494 – 6.055)	**0.002**
**Surgical safety margin**				
≥ 5 mm	Reference		Reference	
< 5 mm or positive	2.967 (1.665 – 5.288)	**< 0.001**	3.063 (1.609 – 5.832)	**0.001**
**Perineural invasion**				
No	Reference		Reference	
Yes	2.079 (1.131 – 3.822)	**0.018**	1.663 (0.801 – 3.451)	0.172
**VI**				
No	Reference		Reference	
Yes	2.238 (1.254 – 4.535)	**0.008**	1.499 (0.692 – 3.247)	0.305
**TC**	0.998 (0.995 – 1.001)	0.283		
**Hemoglobin**	1.002 (0.991 – 1.013)	0.749		
**NLR**	1.041 (0.790 – 1.373)	0.775		
**LMR**	0.974 (0.888 – 1.067)	0.567		
**PAR**	0.995 (0.918 – 1.077)	0.893		
**PLR**	1.000 (0.996 – 1.003)	0.756		
**PNI**	0.989 (0.977 – 1.001)	0.073		
**SIS**				
0	Reference		Reference	
1	1.413 (0.721 – 2.771)	0.791	1.827 (1.326 – 4.466)	0.348
2	2.318 (1.092 – 4.921)	**0.029**	2.141 (1.152 – 5.987)	0.091
**GPS**				
0	Reference			
1–2	1.610 (0.897 – 2.890)	0.110		
**GNRI**				
≥ 94.2	Reference		Reference	
< 94.2	2.056 (1.183 – 3.572)	**0.011**	2.381 (1.306 – 4.341)	**0.005**
**CONUT**				
< 2	Reference		Reference	
≥ 2	2.839 (1.577 – 5112)	**0.001**	2.794 (1.417 – 5.507)	**0.003**
**AJCC stage**				
III	Reference		Reference	
IVa&b	2.334 (1.331 – 4.092)	**0.003**	1.993 (1.096 – 3.626)	**0.024**
**Adjuvant radiotherapy**				
No	Reference		Reference	
Yes	0.320 (0.173 – 0.593)	**< 0.001**	0.327 (0.159 – 0.674)	**0.002**
**Chemotherapy**				
No	Reference			
Yes	0.889 (0.481 – 1.643)	0.707		

*Abbreviations: AJCC* American Joint Committee on Cancer, *CI* confidence interval, *CONUT* Controlling Nutrition scores, *DOI* depth of invasion, *ECOG PS* eastern cooperative oncology group performance status, *ENE* extranodal extension, *GNRI* Geriatric Nutritional Risk Index, *GPS* Glasgow prognostic score, *HR* hazard ratio, *LSCC* lip squamous cell carcinoma, *LMR* lymphocyte-to-monocyte ratio, *NLR* neutrophil-to-lymphocyte ratio, *OS* overall survival, *PAR* platelet-to-albumin ratio, *PLR* platelet-to-lymphocyte ratio, *PNI* prognostic nutrition index, *SIS* systemic inflammation score, *TC* total cholesterol, *VI* vascular invasion

### Establishment the nomograms

**Fig. 2:**
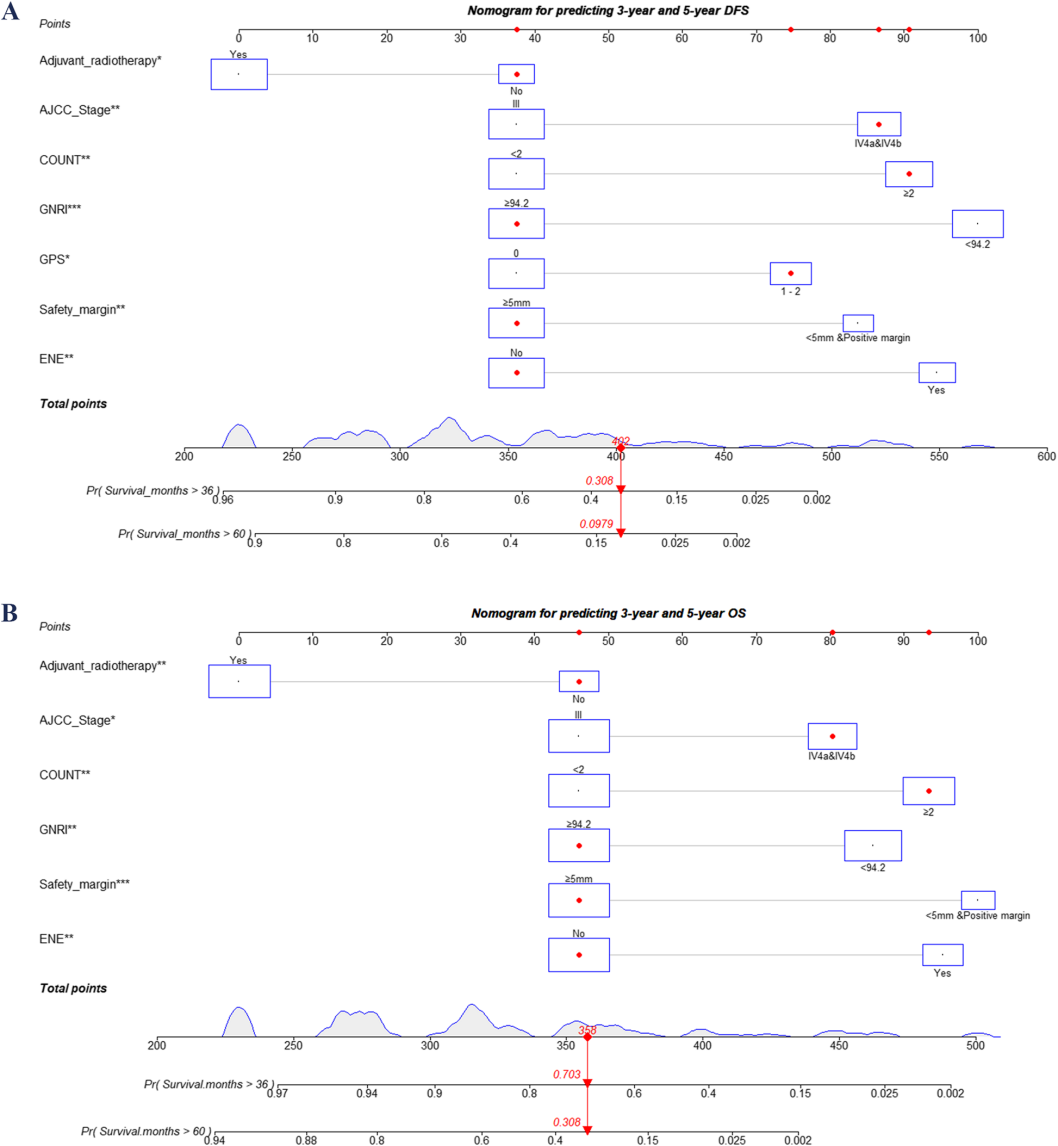
Prognostic nomograms for DFS (**A**) and OS (**B**) in locally advanced LSCC patients post-surgery: a case illustration using new predictive models. This figure demonstrates a patient-specific example utilizing novel models to predict DFS and OS. The size of each rectangle reflects the number of cases corresponding to different prognostic scenarios. AJCC, American Joint Committee on Cancer; COUNT, controlling nutrition scores; DFS, disease-free survival; ENE, extranodal extension; GPS, Glasgow prognostic score; GNRI, Geriatric Nutritional Risk Index; LSCC, lip squamous cell cancer; OS, overall survival

The independent prognostic factors identified through Cox regression analysis were all incorporated into the nomograms that impact DFS and OS. Figure 2 depicts the nomogram models and provides an example of its utilization for predicting 3-year and 5-year DFS and OS.

### Dynamic nomograms

Dynamic nomograms, also referred to as web calculators, possess the advantage of dynamically adjusting prediction results in real-time based on individual patient characteristics, thereby enhancing the accuracy and clinical practicality of personalized treatment decisions. Dynamic nomogram for DFS: https://chenghao66.shinyapps.io/LIP_CANCER_OS/. Dynamic nomogram for OS: https://chenghao66.shinyapps.io/LIP_CANCER_DFS/.

### Validation

**Fig. 3:**
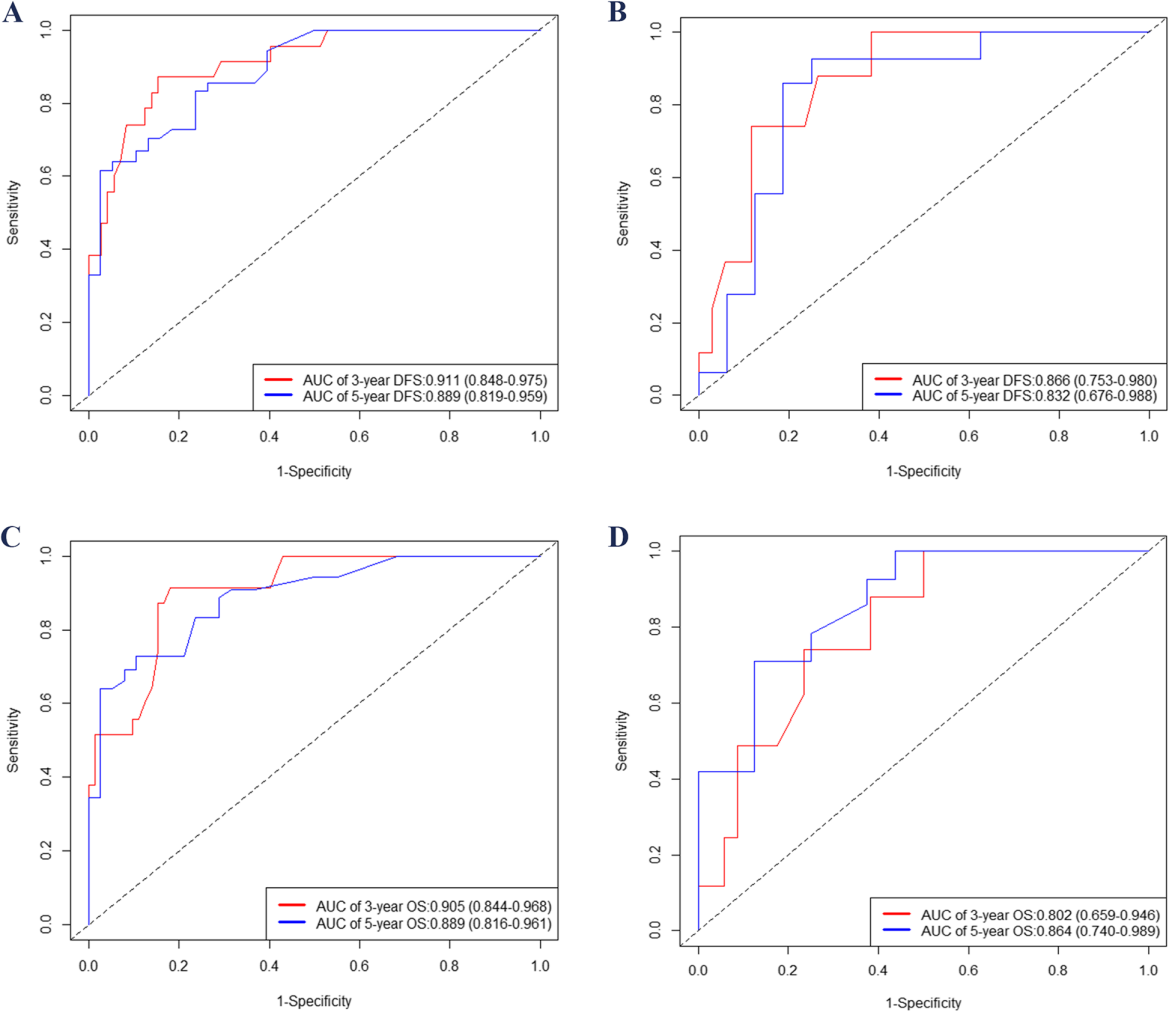
Time-dependent ROC curves. AUC for predicting 3-, and 5-year DFS in the training (**A**) and validation (**B**) set; ROC curves corresponding to 3-, and 5-year OS in the training (**C**) and validation (**D**) cohort. AUC, area under curve; OS, overall survival; DFS, disease-free survival; ROC, receiver operating characteristic

**Fig. 4:**
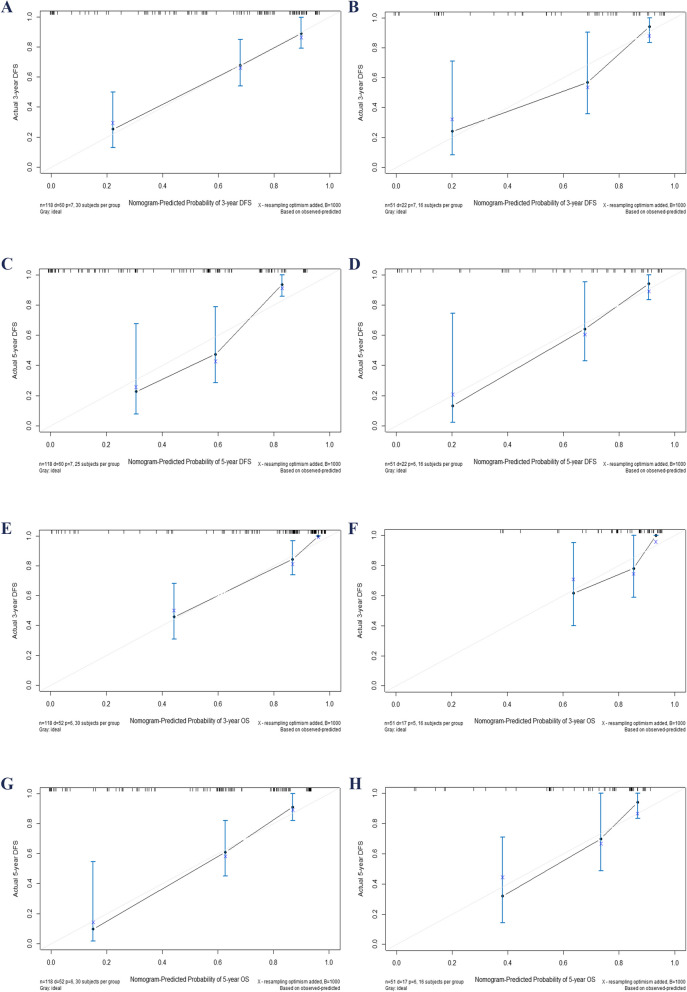
Calibration plots for 3- and 5-year DFS and OS in postoperative locally advanced LSCC patients. This figure presents calibration plots for 3- and 5-year DFS in the training cohort (**A**, **C**) and validation cohort (**B**, **D**), along with OS in the training cohort (**E**, **G**) and validation cohort (**F**, **H**). The X-axis indicates model-predicted survival, while the Y-axis shows actual survival outcomes. The bar line represents the 95% CI based on Kaplan–Meier analysis, with the diagonal line serving as the ideal reference. OS, overall survival; CI, confidence interval; DFS, disease-free survival; LSCC, lip squamous cell cancer

**Fig. 5:**
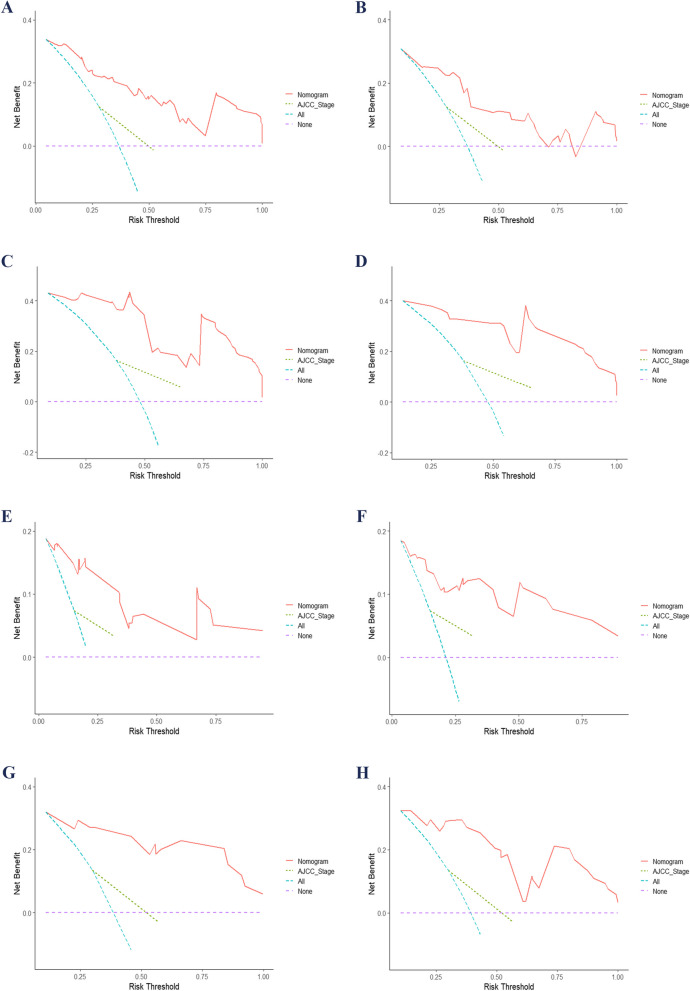
DCA curves comparing nomograms and AJCC staging for DFS and OS. This figure shows DCA curves for 3- and 5-year DFS in the training set (**A**, **C**) and validation set (**B**, **D**), as well as 3- and 5-year OS in the training set (**E**, **G**) and validation set (**F**, **H**). AJCC, American Joint Committee on Cancer; DCA, decision curve analysis; DFS, disease-free survival; OS, overall survival

The ROC curve showed that the model predicted AUC values of 0.911 and 0.889 for the 3-year and 5-year DFS, respectively, in the training group, while in the validation group, these values were 0.866 and 0.832. Similarly, for the 3-year and 5-year OS, the AUC values were found to be 0.905 and 0.889 in the training group, whereas in the validation group, they were observed as 0.802 and 0.864. The ROC curves depicted in Fig. 3, along with their corresponding AUC values, provide compelling evidence of the model's excellent discriminatory ability. The calibration plot in Fig. 4 indicates a good fit, with predicted outcomes closely aligning with the actual observed values. This further confirms that the model exhibits excellent discriminatory capacity and accuracy. Additionally, the Decision Curve Analysis (DCA) curves shown in Fig. 5 highlight that the new nomogram models offer higher net clinical benefits across most threshold intervals compared to the traditional AJCC staging, suggesting that the new model provides a more comprehensive approach. The NRI, IDI and C-index were calculated to evaluate the enhancement of the new model in comparison with the AJCC staging system (Table 4).

**Table 4:** The IDI, NRI, and C-index of the nomograms and AJCC stage system for DFS and OS prediction

	Training cohort			Validation cohort		
	Estimate	95% CI	*P*	Estimate	95% CI	*P*
**IDI (vs. AJCC stage)**						
For 3-year DFS	0.283	0.173–0.429	**< 0.001**	0.261	0.094–0.514	**< 0.001**
For 5-year DFS	0.337	0.205–0.465	**< 0.001**	0.301	0.007–0.528	**0.039**
For 3-year OS	0.312	0.208–0.475	**< 0.001**	0.170	0.027–0.500	**< 0.001**
For 5-year OS	0.339	0.229–0.467	**< 0.001**	0.130	0.042–0.392	**< 0.001**
**NRI (vs. AJCC stage)**						
For 3-year DFS	0.439	0.266–0.644	**< 0.001**	0.409	0.157–0.707	**< 0.001**
For 5-year DFS	0.673	0.407–0.806	**< 0.001**	0.498	0.101–0.810	**0.020**
For 3-year OS	0.528	0.338–0.747	**< 0.001**	0.432	0.022–0.692	**< 0.001**
For 5-year OS	0.679	0.372–0.821	**< 0.001**	0.359	0.040–0.715	**< 0.001**
**C-index**						
The nomogram (DFS)	0.815	0.762–0.868		0.873	0.795–0.951	
The nomogram (OS)	0.817	0.762–0.872		0.759	0.566–0.870	
The AJCC Stage (DFS)	0.605	0.534–0.676		0.677	0.575–0.779	
The AJCC Stage (OS)	0.614	0.540–0.688		0.647	0.520–0.774	
**C-index improvement (vs. AJCC stage)**						
For DFS prediction	0.210		**< 0.001**	0.196		**< 0.001**
For OS prediction	0.203		**< 0.001**	0.112		**< 0.001**

*Abbreviations: AJCC* American joint committee on cancer, *CI* confidence interval, *C-index* concordance index, *DFS* disease-free survival, *IDI* integrated discrimination improvement, *NRI* net reclassification index, *OS* overall survival

### Risk stratification system

**Fig. 6:**
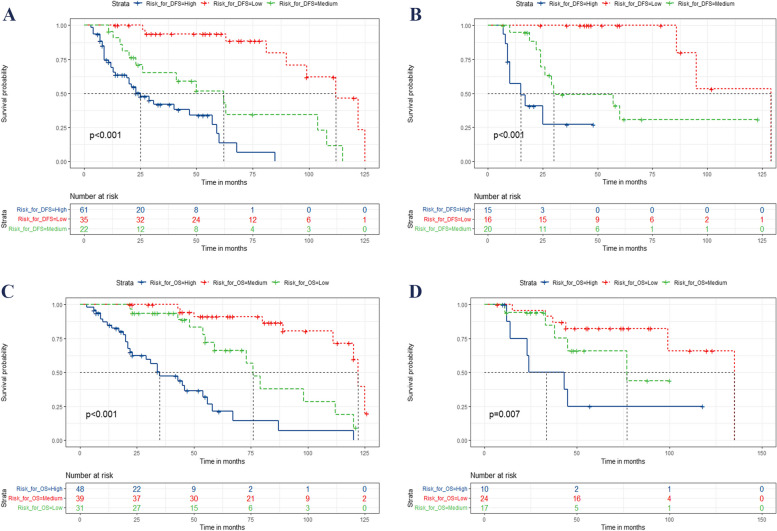
Kaplan–Meier curves for locally advanced LSCC patients post-surgery in training and validation cohort according to the new risk stratification system. Kaplan–Meier curves based on new risk stratification system for predicting DFS (**A**, **C**) and OS (**B**,** D**) in training and validation set, respectively. The red, green, and blue curves represent low-, medium-, and high-risk groups, respectively. DFS, disease-free survival; LSCC, lip squamous cell cancer; OS, overall survival

The X-tile software determined the optimal cut-off point, while the nomogram calculated the total score to classify all patients into three risk subgroups for predicting DFS: high risk (≥ 165.1 points), medium risk (92.1–165.0 points), and low risk (≤ 92.0 points). Similarly, scores of high risk (≥ 160.1), medium risk (100.0–159.5), and low risk (≤ 99.6) were used to predict OS. Kaplan–Meier survival curves demonstrated significant differences in DFS and OS among these three risk subgroups in both the training and validation sets, as illustrated in Fig. 6.

## Discussion

LSCC is among the most common types of oral cancer [6, 20, 21]. The majority of cases, approximately 90%, are observed in the lower lip [22]. Despite the relatively low mortality rate of LSCC, these carcinomas are prone to cause considerable damage through local invasion or metastasis to cervical lymph nodes [23]. The 8th edition of the AJCC staging system is widely recognized as the primary tool for prognostication in LSCC [24]. However, this system does not consider the influence of inflammation and nutrition-related markers on prognosis. The present study investigates the impact of various clinicopathological factors, including inflammation-nutrition-related markers, on the prognosis of locally advanced LSCC.

The CONUT was initially proposed by J. Ignacio de Ulibarri et al. in 2005, and it is derived from the lymphocyte count, serum albumin, and TC, with corresponding scores assigned to each parameter [25]. A decreased lymphocyte count generally indicates immune system suppression, which may facilitate tumor cells evading immune surveillance and promoting tumor progression. Conversely, an elevated lymphocyte count is often associated with enhanced immune response and improved clinical prognosis. Numerous studies have validated the preoperative lymphocyte count, demonstrating a strong correlation with the prognosis of various malignant tumors [26–29]. The integral nutrient reserve, albumin, plays a pivotal role in essential biological processes, such as regulating immune functions and maintaining fluid balance [30]. The serum albumin level serves as an indicator of the inflammatory and nutritional status in patients with malignant tumors [31]. In head and neck cancer individuals undergoing surgery, a low serum albumin level is indicative of a higher likelihood of postoperative complications and an unfavorable prognosis [32, 33]. Cholesterol metabolism promotes tumor growth by reprogramming metabolism and immune responses [34]. The dysregulation of cholesterol metabolism may exert a pivotal influence on the progression of cancer [35]. Meanwhile, the pretreatment serum TC level has also been demonstrated to exhibit a significant correlation with prognosis in certain types of tumors [36–39]. Based on the findings from previous studies, it is reasonable to assert that CONUT plays a crucial prognostic role in patients with malignant tumors, as evidenced by investigations conducted across various tumor sites [40–44].

The GNRI is a widely utilized nutritional screening tool in clinical practice. It primarily focuses on measuring serum albumin concentration and body weight. Due to its straightforward calculation method and user-friendly operation, the GNRI holds significant practicality in daily clinical work by providing an intuitive and effective assessment of a patient's nutritional status while aiding clinicians in identifying potential malnutrition risks. Initially introduced by Olivier Bouillanne and his team in 2005, the GNRI was originally designed to evaluate the nutritional status of hospitalized elderly patients and predict complications associated with malnutrition [45]. With the simple measurement of serum albumin concentration and body weight, the Geriatric GNRI can effectively serve as a reliable indicator of nutritional status, particularly in cancer patients and elderly populations. This invaluable tool aids clinicians in accurately assessing patients' nutritional risk and devising appropriate treatment plans. As research progresses, GNRI is increasingly being applied across various clinical domains, emerging as a pivotal instrument for malnutrition assessment. Its application has expanded to encompass diverse age groups and patients with different types of malignant tumors [46–48], excluding solely elderly patients. The advancement of research has significantly propelled the widespread adoption of this valuable application.

In addition, the GPS is a straightforward assessment tool that utilizes serum albumin levels and C-reactive protein concentrations. It is widely employed for predicting the survival prognosis of cancer patients, particularly in evaluating tumor-related inflammation and nutritional status. Numerous studies have validated the predictive role of GPS in patients with oral cancer, which aligns with our study findings [49–51]. It was observed that patients with lower GPS values exhibited more favorable prognoses. Our research showed a significant correlation between preoperative GPS and DFS in patients diagnosed with locally advanced LSCC.

However, to date, no studies have investigated the relationship between inflammation-nutrition-related markers and the prognosis of locally advanced LSCC. Our study represents the pioneering effort in analyzing the prognostic impact of inflammation-nutrition-related markers such as CONUT, GNRI, and GPS on locally advanced LSCC. Furthermore, we identified CONUT, GNRI, and GPS as independent prognostic factors. This study addresses a significant gap in the current literature by providing novel insights into the potential value of these markers for prognostic evaluation in LSCC patients. Additionally, our subsequent endeavor to incorporate CONUT, GNRI, and GPS into prognostic nomograms is also groundbreaking.

ENE is a significant determinant impacting the staging and prognosis of locally advanced LSCC, as it correlates with an elevated risk of poorer survival. The presence of ENE in cervical lymph node metastasis is significantly associated with a poor prognosis in oral cancer [52]. In the 8th edition of the oral carcinoma staging manual, the AJCC included ENE as a modifier to the N staging [53]. Specifically, the presence of ENE signifies cancer cells infiltrating lymph nodes beyond their surrounding capsule, resulting in heightened biological aggressiveness. This characteristic can substantially influence the prognosis of LSCC patients, particularly concerning disease recurrence, distant metastasis, and survival outcomes. Given that ENE typically indicates a poorer prognosis, clinical management strategies necessitate adjustment [54].

In the management of LSCC, the surgical margin plays a crucial role in determining patient prognosis. The resection margin status is typically categorized as positive (indicating residual cancer) or negative (indicating no residual cancer), and directly impacts the risk of recurrence, survival rates, and subsequent treatment decisions. A study led by Brinkman David et al. identified a postoperative margin of less than 3 mm as an independent prognostic factor in oral squamous cell carcinoma (OSCC) patients [55]. The findings of other studies have also demonstrated that the optimal safety margin for OSCC should exceed 5 mm, and cases with a safety margin of less than 5 mm exhibit significantly worse prognosis [55, 56]. The results of another retrospective study involving 147 LSCC patients demonstrated that individuals with surgical safe margins ≥ 5 mm exhibited significantly improved survival outcomes [57]. Our study demonstrated that patients with a surgical margin ≥ 5 mm exhibited significantly improved prognostic outcomes, which is similar to the results of previous studies.

According to clinical guidelines, the primary treatment for locally advanced LSCC consists of surgical intervention followed by adjuvant radiotherapy [54, 58, 59]. Our study yielded similar findings, demonstrating significantly enhanced DFS and OS in patients who received postoperative adjuvant radiotherapy compared to those who did not receive it. Furthermore, previous studies have indicated that postoperative concurrent chemoradiotherapy provides superior survival benefits when ENE is present and/or the surgical safe margin < 1 mm [60]. However, our analysis suggests that administering chemotherapy concurrently with radiotherapy does not enhance survival outcomes. This finding may be attributed to the fact that not all patients in our study met these criteria for receiving postoperative concurrent chemoradiotherapy or due to the limited number of cases included in our study. The possibility of future studies with targeted designs is not ruled out.

The Tumor-Node-Metastasis (TNM) staging system was first introduced by French surgeon Pierre Denoix in 1953 and remains the gold standard for prognostic evaluation of tumors [61]. The most recent iteration is the 8th edition of the AJCC staging system [62, 63]. However, the TNM system has certain limitations [64]. Firstly, this system primarily categorizes disease stages based on anatomical structure while neglecting several key factors that may influence prognosis. Consequently, patients with similar anatomical characteristics are classified into the same stage despite exhibiting different outcomes (e.g., recurrence or survival), leading to clinical heterogeneity. Secondly, the TNM staging treats the primary tumor, lymph node involvement, and metastases as discrete variables rather than continuous ones, which restricts the granularity of stage classification and complicates individualized prognosis assessment. Additionally, patients within the same stage may exhibit markedly different prognoses. In contrast, the application of the nomogram in this study offers a more transparent and accurate prognosis assessment, particularly for patients with locally advanced LSCC following surgical treatment. Compared to the TNM staging system, the nomogram provides a more personalized prediction by integrating specific patient characteristics and disease factors, thereby enhancing the precision of the prognosis assessment [64, 65]. A significant advantage of nomograms is their capability to estimate individualized prognostic risk based on patient-specific characteristics and disease-related factors [66]. Unlike the traditional TNM system, nomograms can incorporate both continuous variables and multiple disease-related factors, thereby offering a more precise and nuanced prognosis assessment. Consequently, nomograms provide a more comprehensive and personalized reflection of patient outcomes, offering valuable insights for clinical decision-making. Additionally, by integrating various prognostic factors, nomograms address the limitations of the TNM staging system and deliver more detailed prognostic information to patients. In this study, the nomogram effectively illustrated the post-surgical prognosis for patients with locally advanced LSCC. Through the calculation of the IDI, NRI, and C-index, we determined that the nomogram model exhibits superior performance compared to the 8th edition of the AJCC staging system in prognostic prediction.

This study possesses several strengths and limitations. It is notably innovative as it is the first to investigate the correlation between preoperative inflammation-nutrition-related markers and postoperative prognosis of locally advanced LSCC. Furthermore, it introduces CONUT, GNRI, and GPS into the prognostic model of LSCC for the first time, demonstrating superior prognostic accuracy than the traditional AJCC staging system. However, there are some limitations to this study. The exclusion of patients who died within 30 days post-surgery may lead to selection bias. This could result in a study population that is disproportionately comprised of healthier individuals with better surgical tolerance. Consequently, the overall prognosis of the study cohort may appear more favorable than that of real-world patients, limiting the external validity of the results. Additionally, the relatively small sample size may restrict the generalizability of the findings. Several key prognostic factors, such as comorbidities, molecular biomarkers, and lifestyle variables, were also not included in the analysis.Future studies should address these limitations by conducting larger prospective multicenter studies and incorporating multi-omics data to strengthen and enhance the comprehensiveness of prognostic models.

## Conclusion

The presence of GPS, CONUT, and GNRI significantly influences the prognosis of patients with locally advanced LSCC after surgery. By incorporating these inflammation-nutrition-related markers alongside other clinicopathological factors, the nomogram demonstrates superior predictive efficacy and provides greater net clinical benefit compared to the AJCC staging system.

## Supplementary Information

Supplementary Material 1.

Supplementary Material 2.

Supplementary Material 3.

## Data Availability

The data supporting this study are available upon reasonable request from the corresponding author.

## Abbreviations

AJCC: American Joint Committee on Cancer
AUC: Area under the curve
BMI: Body mass index
C-index: Concordance index
CI: Confidence interval
CONUT: Controlling Nutritional status
CRT: Conformal radiotherapy
DFS: Disease-free survival
DCA: Decision curve analysis
DOI: Depth of invasion
ECOG: Eastern cooperative oncology group
ENE: Extranodal extension
GPS: Glasgow prognostic score
GNRI: Geriatric Nutritional Risk Index
LMR: Lymphocyte-to-monocyte ratio
LSCC: Lip squamous cell carcinoma
IMRT: Intensity-modulated radiotherapy
IDI: Integrated discrimination improvement
IQR: Interquartile range
NLR: Neutrophil-to-lymphocyte ratio
NRI: Net reclassification improvement
OS: Overall survival
OSCC: Oral squamous cell carcinoma
PAR: Platelet-to-albumin ratio
PLR: Platelet-to-lymphocyte ratio
PNI: Prognostic nutrition index
PS: Performance status
ROC: Receiver operating characteristic
SIS: Systemic inflammation score
TC: Total cholesterol
TNM: Tumor-Node-Metastasis
VI: Vascular invasion
VIF: Variance inflation factor
VMAT: Volumetric-modulated arc therapy
