# Supplementary material for: Development of a predictive nomogram based on preoperative inflammation-nutrition-related markers for prognosis in locally advanced lip squamous cell carcinoma after surgical treatment
Source: BMC Oral Health. 2025 Feb 20;25:268. doi: 10.1186/s12903-025-05663-6 (PMC11843749; doi:10.1186/s12903-025-05663-6)
Supplement: Supplementary file 1 — Supplementary Material 1. [file 12903_2025_5663_MOESM1_ESM.docx]

| **Table S1** The formula for calculating CONUT. | | |
| --- | --- | --- |
| Paramater | Range | Score |
| Serum Albumin (g/L) | ≥ 35 | 0 |
|  | 30–34 | 2 |
|  | 25-29 | 4 |
|  | <25 | 6 |
| Lymphocyte Count (10^9^/L) | ≥ 1.6 | 0 |
|  | 1.2–1.599 | 1 |
|  | 0.8–1.199 | 2 |
|  | < 0.8 | 3 |
| Total Cholesterol (mg/dL) | ≥ 180 | 0 |
|  | 140–179 | 1 |
|  | 100–139 | 2 |
|  | < 100 | 3 |
| *Abbreviation* CONUT, controlling nutritional status. | | |
