# Supplementary material for: Development of a predictive nomogram based on preoperative inflammation-nutrition-related markers for prognosis in locally advanced lip squamous cell carcinoma after surgical treatment
Source: BMC Oral Health. 2025 Feb 20;25:268. doi: 10.1186/s12903-025-05663-6 (PMC11843749; doi:10.1186/s12903-025-05663-6)
Supplement: Supplementary file 2 — Supplementary Material 2. [file 12903_2025_5663_MOESM2_ESM.docx]

| **Table S2** Calculation formulas in this study. | |
| --- | --- |
| **Clinical index** | **Calculation formula** |
| GPS | Score of 0 if C-reactive protein ≤ 10 mg/dL and albumin ≥ 35 g/L  Score of 1 if C-reactive protein > 10 mg/dL or albumin < 35 g/L  Score of 2 if C-reactive protein > 10 mg/dL and albumin < 35 g/L |
| BMI | Mass (kg) / height^2^(m^2^) |
| SIS | Albumin < 40 g/L & LMR < 4.44 is scored as 2; Albumin ≥ 40 g/L or LMR ≥ 4.44 is scored as 1; Albumin ≥ 40 g/L & LMR ≥ 4.44 is scored as 0 |
| LMR | Lymphocyte count / monocyte count |
| PAR | Platelet count / albumin (g/L) |
| PNI | Serum albumin (g/L) + (lymphocyte count ×5) |
| NLR | Neutrophil count / lymphocyte count |
| PLR | Platelet count / lymphocyte count |
| GNRI | 1.489 × albumin (g/L) + 41.7 × current body weight (kg)/ ideal body weight (kg). If the current weight exceeds the ideal weight, the ratio is capped at one. The ideal body weight = 22 × height (m^2^), where 22 represents the standard BMI for optimal health. |
| *Abbreviations* BMI, body mass index; GPS, Glasgow prognostic score; PLR, platelet-to-lymphocyte ratio; PNI, prognostic nutrition index; NLR, neutrophil-to-lymphocyte ratio; SII; systemic immune-inflammation index. | |
