# Supplementary material for: Development of a predictive nomogram based on preoperative inflammation-nutrition-related markers for prognosis in locally advanced lip squamous cell carcinoma after surgical treatment
Source: BMC Oral Health. 2025 Feb 20;25:268. doi: 10.1186/s12903-025-05663-6 (PMC11843749; doi:10.1186/s12903-025-05663-6)
Supplement: Supplementary file 3 — Supplementary Material 3. [file 12903_2025_5663_MOESM3_ESM.docx]

| **Table S3** Tolerance and VIF Values of Variables Included in the Analysis. | | | | | |
| --- | --- | --- | --- | --- | --- |
|  | **Tolerance** | **VIF** |  | **Tolerance** | **VIF** |
| **Gender** | 0.744 | 1.345 | **NLR** | 0.801 | 1.249 |
| **Age at diagnosis** | 0.810 | 1.234 | **LMR** | 0.680 | 1.417 |
| **Tumor location** | 0.816 | 1.226 | **PAR** | 0.819 | 1.221 |
| **Differentiation** | 0.851 | 1.400 | **PLR** | 0.722 | 1.385 |
| **DOI** | 0.749 | 1.335 | **PNI** | 0.257 | 3.898 |
| **Smoking** | 0.709 | 1.411 | **SIS** | 0.526 | 1.902 |
| **ECOG PS score** | 0.733 | 1.365 | **GPS** | 0.352 | 2.841 |
| **ENE** | 0.342 | 2.924 | **GNRI** | 0.292 | 3.421 |
| **Surgical safety margin** | 0.257 | 3.894 | **CONUT** | 0.237 | 4.221 |
| **Perineural invasion** | 0.740 | 1.352 | **AJCC stage** | 0.283 | 3.539 |
| **VI** | 0.643 | 1.555 | **Adjuvant** **radiotherapy** | 0.499 | 2.006 |
| **TC** | 0.345 | 2.897 | **Chemotherapy** | 0.322 | 3.105 |
| **Hemoglobin** | 0.805 | 1.242 |  |  |  |
| *Abbreviations* AJCC, American Joint Committee on Cancer; CONUT, controlling nutrition scores; DOI, depth of invasion; ECOG PS, eastern cooperative oncology group performance status; ENE, extranodal extension; GNRI, Geriatric Nutritional Risk Index; GPS, Glasgow prognostic score; LMR, lymphocyte-to-monocyte ratio; NLR, neutrophil-to-lymphocyte ratio; PAR, platelet-to-albumin ratio; PLR, platelet-to-lymphocyte ratio; PNI, prognostic nutrition index; SIS, systemic inflammation score; TC, total cholesterol; VI, vascular invasion; VIF, variance inflation factor. | | | | | |
